# The detection of left ventricular scar by delayed enhancement-CMR in non-ischemic cardiomyopathy is a stronger predictor of cardiovascular events than left ventricular ejection fraction

**DOI:** 10.1186/1532-429X-15-S1-O95

**Published:** 2013-01-30

**Authors:** Carlos M Orrego, Andrea M Cordero-Reyes, Mohamad G Ghosn, Jerry D Estep, Guillermo Torre-Amione, Sven Zuehlsdorff, Gary R McNeal, Dipan J Shah

**Affiliations:** 1 Cardiology, The Methodist Hospital, Houston, TX, USA; 2 Cardiology, Weill Cornell Medical College, Houston, TX, USA; 3 Baylor College of Medicine, Houston, TX, USA; 4 Siemens, Houston, TX, USA

## Background

Left ventricular ejection fraction (LVEF) is a strong predictor of subsequent cardiovascular events in patients with ischemic and non-ischemic cardiomyopathy. CMR is an excellent technique for assessment of LVEF as well as for the detection of myocardial scar. We sought to evaluate if the presence of myocardial scarring in non-ischemic cardiomyopathy could be an important predictor of cardiovascular events.

## Methods

Inclusion criteria were absence of coronary artery disease, a diagnosis of cardiomyopathy and an LVEF ≤ 45%. Patients with a diagnosis of infiltrative disorders, hypertrophic cardiomyopathy, or tachycardia-induced cardiomyopathy were excluded. A total of 132 patients (with greater than 12 months follow up) were analyzed. Imaging was performed on a 1.5 Tesla Avanto, or 3.0 Tesla Verio MRI scanner (Siemens Medical Solutions). Cine images were performed using a steady state free precession pulse sequence. Delayed enhancement (DE) CMR was performed 10 minutes after administration of 0.15 mmol/kg Gadopentetate Dimeglumine (Bayer HealthCare) with typical in-plane resolution 18 x 2.0 mm, slice thickness 6-7 mm. LV and RV volumes and EF were analyzed by manual planimetry of endocardial contours during end-diastole and end-systole. DE-CMR images were analyzed to identify hyperenhanced myocardium (> 2 SD of remote signal intensity). The primary outcome was a combination of hospitalization for heart failure, LV assist device (LVAD) implantation, cardiac transplant, or death. Adjusted Kaplan-Meier curves were constructed for groups with and without scar by CMR with p<0.05 considered significant. Multivariate logistic regression analysis was performed.

## Results

Patient's demographics are shown in table. All baseline demographic data were similar between patients with and without scar by CMR. The adjusted event-free survival rate was significantly lower for patients with scar compared to patients without scar by CMR at 2-years (p=0.03) (graph). Mean scar burden was 3 ± 8% of LV. On multivariable analysis, the presence of scar was an independent predictor of events, HR 2.5(CI: 0.98-6.38, p=0.04), but LVEF was not.

**Table 1:** Patient Characteristics

	All	Without Scar	With scar	p value
Patients (n)	132	65	67	

Clinical characteristics				

Age (years)	51 ± 12	51 ± 16	50 ± 12	0.7

BMI (kg/m2)	31 ± 9	30 ± 8	32 ± 9	0.6

Male gender (%)	75(57%)	32(49%)	43(64%)	0.06

Race				

Caucasian (%)	70(53%)	36(56%)	34(51%)	0.8

Black (%)	35(27%)	17(26%)	18(27%)	0.9

Hispanic (%)	17(12%)	10(15%)	7(10%)	0.8

Other (%)	10(8%)	2(3%)	8(12%)	0.08

DM (%)	26(20%)	15(23%)	16(24%)	0.9

HTN (%)	45(34%)	26(40%)	29(43%)	0.5

CMR findings				

LVEF(%)	31± 11%	33± 10	30± 10	0.09

Scar burden	3 ± 5	N/A	6 ± 6	N/A

## Conclusions

In non-ischemic cardiomyopathy the presence of LV scar measured by DE-CMR is a stronger predictor of cardiovascular events than LVEF.

## Funding

Research grant from Siemens.

**Figure 1:**
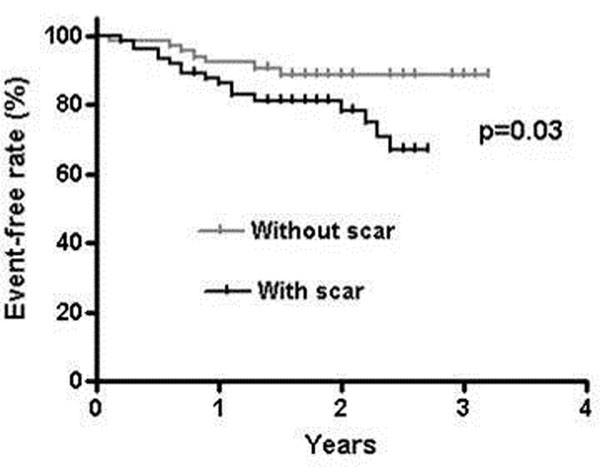
Event-free survival in patients with and without scar by CMR

